# Surgery versus Watchful Waiting in Patients with Craniofacial Fibrous Dysplasia – a Meta-Analysis

**DOI:** 10.1371/journal.pone.0025179

**Published:** 2011-09-23

**Authors:** Moran Amit, Michael T. Collins, Edmond J. FitzGibbon, John A. Butman, Dan M. Fliss, Ziv Gil

**Affiliations:** 1 Department of Otolaryngology Head and Neck Surgery, Tel Aviv Sourasky Medical Center, Sackler Faculty of Medicine, Tel Aviv University, Tel Aviv, Israel; 2 Skeletal Clinical Studies Unit, Craniofacial and Skeletal Diseases Branch, National Institute of Dental and Craniofacial Research, National Institutes of Health, Bethesda, Maryland, United States of America; 3 National Eye Institute, National Institutes of Health, Bethesda, Maryland, United States of America; 4 Department of Diagnostic Radiology, National Institutes of Health, Bethesda, Maryland, United States of America; 5 Head and Neck Surgery Unit and the Laboratory for Applied Cancer Research, Tel Aviv Sourasky Medical Center, Sackler Faculty of Medicine, Tel Aviv University, Tel Aviv, Israel; Harvard Medical School, United States of America

## Abstract

**Background:**

Fibrous dysplasia (FD) is a benign bone tumor which most commonly involves the craniofacial skeleton. The most devastating consequence of craniofacial FD (CFD) is loss of vision due to optic nerve compression (ONC). Radiological evidence of ONC is common, however the management of this condition is not well established. Our objective was to compare the long-term outcome of patients with optic nerve compression (ONC) due to craniofacial fibrous dysplasia (CFD) who either underwent surgery or were managed expectantly.

**Methodology/Principal Findings:**

We performed a meta-analysis of 27 studies along with analysis of the records of a cohort of patients enrolled in National Institutes of Health (NIH) protocol 98-D-0145, entitled Screening and Natural History of Fibrous Dysplasia, with a diagnosis of CFD. The study group consisted of 241 patients; 122 were enrolled in the NIH study and 119 were extracted from cases published in the literature. The median follow-up period was 54 months (range, 6–228 months). A total of 368 optic nerves were investigated. All clinically impaired optic nerves (n = 86, 23.3%) underwent therapeutic decompression. Of the 282 clinically intact nerves, 41 (15%) were surgically decompressed and 241 (85%) were followed expectantly. Improvement in visual function was reported in fifty-eight (67.4%) of the clinically impaired nerves after surgery. In the intact nerves group, long-term stable vision was achieved in 31/45 (75.6%) of the operated nerves, compared to 229/241 (95.1%) of the non-operated ones (p = 0.0003). Surgery in asymptomatic patients was associated with visual deterioration (RR 4.89; 95% CI 2.26–10.59).

**Conclusions:**

Most patients with CFD will remain asymptomatic during long-term follow-up. Expectant management is recommended in asymptomatic patients even in the presence of radiological evidence of ONC.

## Introduction

Fibrous dysplasia (FD) is a benign, slowly growing fibro-osseous disease accounting for 5–10% of all bone tumors. The skull base is the most common site of involvement in the craniofacial skeleton [1]. The pathophysiologic process involves the replacement of normal bone with immature bone marrow stromal cells whose differentiation was arrested due to somatic missense mutations in the gene *GNAS* on chromosome 20 [2],[3]. The disease can involve a single bone (monostotic variant), multiple bones (polyostotic variant), or present in combination with hyperfunctioning endocrinopathies (e.g. precocious puberty and hyperthyroidism) known as the McCune Albright syndrome (MAS) [4], [5]. The symptoms associated with FD are well described in the literature, with some series reporting up to one-third of patients having visual complaints, including blurred vision, diplopia, and epiphora [6]. The most devastating consequence of CFD is the loss of vision due to optic nerve compression (ONC). Although radiological evidence of ONC in CFD is found in 50–90% of the patients [6], the management of these patients is more often based on personal experience and/or expert opinion.

Earlier reports on a cohort of patients from a single center who presented for medical treatment by Lee et al and Cutler et al showed that most ONC is asymptomatic. However the natural history of CFD that includes subjects reporting for surgical management is not well documented [6], [7]. Here we report the long-term visual outcome of patients who had either undergone surgery or were managed expectantly. Towards this end, we reviewed the clinical records of FD patients seen at the National Institutes of Health (NIH) since 1998 together with all published cases found in the literature. A meta-analysis performed revealed better results in patients managed expectantly than those undergoing prophylactic decompression.

## Methods

### Objective

To compare the long-term outcome of patients with optic nerve compression (ONC) due to craniofacial fibrous dysplasia (CFD) who either underwent surgery or were managed expectantly.

### Participants

The records of all patients diagnosed as having CFD and who were enrolled in National Institutes of Health (NIH) protocol 98-D-0145, entitled Screening and Natural History of Fibrous Dysplasia, seen between 1998 and 2010 were evaluated. The diagnosis of CFD was made based on the results of clinical, radiographic, and histological studies. CFD was identified by computer tomography (CT) analyses. All patients underwent testing of all relevant endocrine axes. A diagnosis of growth hormone (GH) excess was made on the basis of a serum GH level >1.0 ng/ml measured 60 minutes after a standard oral glucose tolerance test. Each study patient had a neuro-ophthalmologic evaluation by the same neuro-ophthalmologist (EJF) that included best-corrected visual acuity, according to the Early Treatment Diabetic Retinopathy Study scale (20/20 denotes perfect vision), visual fields obtained with the Humphrey Visual Field Analyzer (Humphrey Instruments, San Leandro, CA) using the Swedish Interactive Thresholding Algorithm (SITA) 30–2 program or Goldmann perimetry testing, color vision by 14 Ishihara color plates, contrast sensitivity testing using the Pelli Robson charts, and funduscopy. Abnormalities suggestive of optic neuropathy were defined as either an abnormal result on the visual field test (such as scotoma or field deficit) or an abnormal result on two of the three other tests performed (specifically, visual acuity <20/40, correct identification of <10 of 14 Ishihara color plates, or evidence of optic atrophy on funduscopy). All patients underwent standardized CT imaging of the cranium on either a 4- or 8-slice helical scanner, with a slice reconstruction interval of 1.25–1.50 mm. The patients' demographic and clinical data are summarized in **table 1**.

**Table 1:** Demographic and clinical data of patients seen at the National Institutes of Health (NIH).

	Total† (n = 122)	Asymptomatic patients without surgery(n = 108)	Asymptomatic patients undergoing surgery(n = 8)	Symptomatic patients undergoing surgery(n = 11)	p Value of asymptomatic, observation vs surgery	p Value of, symptomatic vs asymptomatic
Age (years)*	20±17	21±18	20±13	14±5	0.15	1.41
Male/female	50/72	45/63	4/4	4/7	0.71	0.6577
Polyostotic/MAS	18/104	16/92	2/6	2/9	0.6	0.16
Craniofacial pain	50 (41%)	46 (43%)	6 (75%)	6 (55%)	0.13	0.63
Cosmetic deformity	84 (69%)	72 (67%)	7 (88%)	10 (91%)	0.43	1
Café o lait	85 (70%)	73 (68%)	6 (75%)	9 (82%)	1	1
Precocious puberty	61 (50%)	53 (49%)	3 (38%)	7 (64%)	0.71	0.36
Thyroid abnormalities	83 (68%)	73 (68%)	6 (75%)	9 (82%)	1	1
Growth hormone excess	28 (23%)	22 (20%)	4 (50%)	5 (45%)	0.07	1
Follow-up years	8.4±3.37	8.4±3.3	9.6±3.5	7.4±4.3	0.98	1.18

*Age at date of NIH enrollment, mean ± standard deviation.

† Five patients had different management for each eye and were counted twice.

MAS = McCune Albright syndrome.

### Meta analysis search strategy and selection criteria

**Figure 1:**
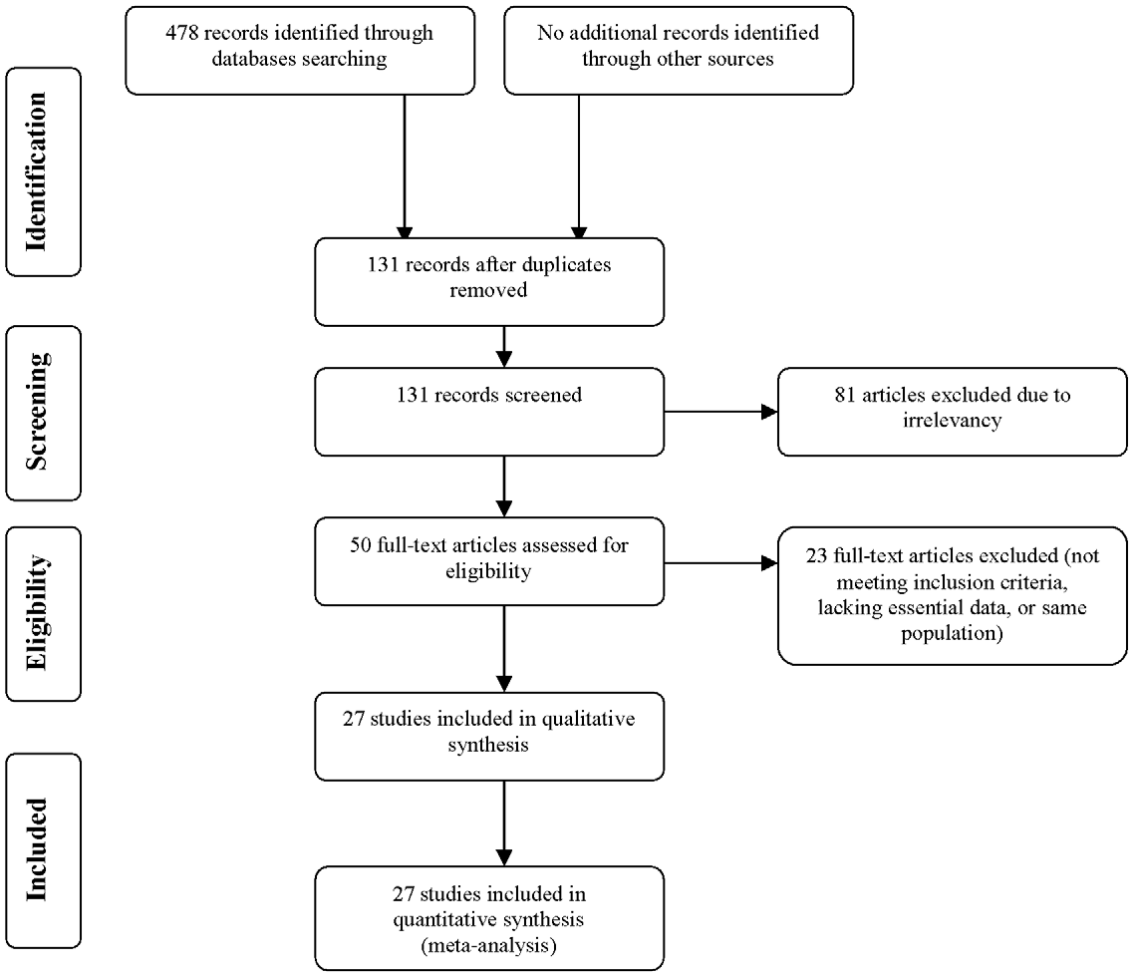
Flow chart of the study selection process.

During October 2010, we conducted a systematic electronic literature database search of PubMed, CINAHL, Cochrane Central Register of Clinical Trials, Cochrane Database of Systematic Reviews and Google from 1975 to 2010. The searches were conducted using the Medical Subject Heading (MeSH) terms (fibrous dysplasia) AND (orbit OR vision OR visual OR optic OR exophthalmos OR diplopia OR proptosis) and limited to “Human”. Reference lists of retrieved manuscripts were hand-searched for additional publications. Eighteen publications in a language other than English that could not be translated because of resource constraints were excluded. Two reviewers (M.A. and Z.G.) independently screened all titles available as well as abstracts produced by the electronic search strategies. Articles were rejected at the initial screening if titles or abstracts showed that they were clearly irrelevant. The full text of potentially relevant articles was reviewed to assess their suitability for inclusion in this meta-analysis. Study selection process is described in **figure 1**. Inclusion criteria according to the study design were any randomized controlled trial, prospective and retrospective cohorts, case-control study designs, case reports and case series. Criteria for study population inclusion were histopathologic diagnosis of CFD, radiologically confirmed optic canal narrowing, pre- and post-treatment visual status (based on visual fields and visual acuity), and >4 months of follow-up. The included studies are listed in **Tables S1, S2, S3 (supplementary material)** [8], [9], [10], [11], [12], [13], [14], [15], [16], [17], [18], [19], [20], [21], [22], [23], [24], [25], [26], [27], [28], [29], [30], [31], [32], [33]. Follow-up included periodic (6–12 months) radiological imaging with CT and MRI as well as ophthalmologic exams.

### Assessment of study quality and risk of bias

Two independent reviewers assessed both the quality and risk of bias of the included publications. They were not blinded to publication details, but measures were taken to obscure outcome assessment, avoid the description of incomplete outcome data, and assess the risk of selective outcome reporting. All the included studies were rated by the Oxford Centre for Evidence-based Medicine suggested levels of evidence according to the study designs.

### Data abstraction

The two reviewers independently checked each case, and all available specific data and endpoints were recorded for each patient. In addition, the authors of the included articles were contacted by email (when available) for additional information on patient follow-up. Discrepancies between the two reviewers were resolved by discussion and consensus. They were not blinded to publication details, but used identical abstraction forms to record the collected data. Details on the individual patient's gender were not always available. Symptomatic or clinical impairments were defined by the presence of a visual field defect or reduced corrected visual acuity.

### Ethics

The study was conducted according to an institutional review board-approved protocol (National Institutes of Health protocol 98-D-0145) and the participants or their guardians gave a written informed consent.

### Statistical methods

Risk ratios and 95% confidence intervals (CIs) were calculated for the individual endpoints. Clinical heterogeneity was assessed by comparison of effect sizes between symptomatic patients who underwent ON decompression, asymptomatic patients who underwent optic nerve decompression, and asymptomatic patients who were managed expectantly. Differences between these three groups were tested using a standard Fisher Exact test (on two degrees of freedom). A two-sided p value<0•05 was considered statistically significant. An independent T test was performed to compare the means of continuous variables using a 95% CI for the difference. Statistical analyses were performed using the SAS software package (SAS Institute, Cary, North Carolina by a certified statistician). The meta-analysis was performed in compliance with the Preferred Reporting Items for Systematic Reviews and Meta-Analyses (PRISMA) statement [34].

## Results

The study selection process is displayed in **Fig. 1**. Fifty relevant articles were reviewed, of which 20 were excluded due to insufficient data or for inability to meet the inclusion criteria (e.g., lack of essential data, follow-up <4 months). Two publications describing the same study population were excluded [7], and a third one was excluded due to the administration of high-dose biphosphonate treatment [35]. We excluded one symptomatic patient in our cohort with extensive disease that was inoperable. Two patients were considered to be asymptomatic because the visual disturbances described were stable, subtle and did not meet the definition of optic neuropathy.

The final study group consisted of 241 patients: 122 were enrolled in the NIH study and 119 were extracted from published cases in the literature. The FD was classified as being monostotic in 72 (30%) cases and as polyostotic in 50 (21%) cases. MAS was diagnosed in 119 (49%) patients. The median age at CFD presentation was 20 years (range, 8–55 years). The median follow-up period was 54 months (range, 6–228 months).

A total of 368 optic nerves were studied, 127 (35%) of them involved both eyes. All patients with clinically impaired optic nerves (i.e. an objective evidence of visual disturbance) (n = 86 nerves, 23.3%) underwent therapeutic optic nerve decompression. Of the 282 asymptomatic ONC, 41 (15%) were decompressed prophylactically and 241 (85%) were followed expectantly. There was no significant difference in the mean age of the patients in the three study groups: 22±11 years (range 8–55) for the symptomatic patients, 19±7 years (range 11–37) for the asymptomatic operated group, and 21±8 years (range12–26) for the asymptomatic non-operated group (p = 0•36). There was also no difference in the follow-up period for the operated and non-operated cohorts of asymptomatic patients (96±44 and 102±16, respectively; p = 0•57), while the follow-up period for the symptomatic patients cohort was significantly shorter (42±27 months; p<0•001). The distribution of FD among the symptomatic and asymptomatic operated patients was similar (monostotic, polyostotic, and MAS rates of 39, 19, and 17 and 22, 9, and 7%, respectively). There was a significantly higher rate of MAS in the non-operated patients group (n = 95, 73% p<0•0001).

The visual outcome in the three groups revealed that 58 (67.4%) of the clinically impaired nerves that underwent surgical decompression had better postoperative visual function than before surgery. Among those subjects who had normal vision but underwent prophylactic optic nerve decompression, long-term stable vision was achieved in 31 (75.6%) of the operated nerves, compared to 229 (95.1%) of the non-operated nerves (three way ANCOVA, p = 0•0003). Surgery in asymptomatic patients was associated with worse postoperative visual function, and a relative risk of 4.89 (95% CIs of 2.26–10.59) for impaired optic nerve function. The type of CFD (MAS, monostotic or polyostotic variants) or GH access were not associated with postoperative worsening of visual outcome in any of the groups (p>0•05), as shown in **table 2**.

**Table 2:** Visual deterioration by disease type*.

	Therapeutic surgery	Prophylactic surgery	Watchful waiting
Monostotic FD	19/41 (46%)	4/23 (17%)	2/17 (11%)
Polyostotic FD	4/21 (19%)	2/11 (18%)	3/39 (8%)
MAS	10/24 (41%)	2/7 (28%)	6/186 (3%)

*No correlation reached a level of significance.

FD = fibrous dysplasia, MAS = McCune Albright's syndrome.

In order to confirm our results, we repeated the analysis on the 122 patients who were followed in a single institution (NIH). In agreement with our previous findings, there were no significant demographic or clinical differences between the three groups (**table 1**). Statistical analyses of this single institution cohort showed that better visual outcome was achieved in 9 out of 14 (64%) clinically impaired operated nerves after surgery in symptomatic patients. Similar to the findings from the pooled data, long-term stable vision of the non-clinically impaired nerves was achieved in 60% of the operated nerves, and in 97.2% non-operated nerves.

## Discussion

Radiological evidence of optic canal involvement in CFD is relatively common [7]. ONC can result from primary CFD of the sphenoid bone or from secondary lesions, including cystic FD, mucoceles, hemorrhage, and aneurysmal bone cysts [27]. While there is consensus that therapeutic decompression of the optic nerve is indicated for symptomatic patients, the question of whether to operate on asymptomatic patients is an ongoing controversial issue [6], [8], . Earlier studies had reported visual impairment in 20–80% of patients with ONC [19], [23]. Similarly, Chen et al reported that one-third of CFD patients will have visual deficits, and two-thirds will report some degree of visual disturbance if optic canal involvement is demonstrated by imaging [8]. Due to the potential risk of rapidly progressing visual deterioration and irreversible optic nerve atrophy, many studies suggest prophylactic decompression of the nerve in asymptomatic CFD patients with radiologic evidence of optic nerve compression [8], [37], [38]. Contrary to these studies, Lee et al reported in her sentinel study that the vast majority of asymptomatic patients with CFD will demonstrate normal vision despite radiological evidence of optic nerve compression, but this cohort did not include a group managed with optic nerve decompression [6]. In a subsequent study on patients from the same institution that included additional subjects (n = 87), Cutler et al, reported no age-related progression to optic neuropathy in these patients.

In the current study, we analyzed the long term follow up data of the patients from the NIH that now also included information regarding treatment and long term follow up (n = 122). In addition, we performed a meta-analysis of all reported cases of patients with radiologic evidence of optic nerve compression. A total of 368 nerves were included in our study, and long-term follow-up of their visual outcome revealed that surgery in asymptomatic patients is associated with worse prognosis compared to the outcome of asymptomatic patients who are managed expectantly. These results are further supported by the similar results that emerged from our analysis of the data from a single institution.

The majority of the patients in our cohort had MAS. In the current analysis, there was not a significant difference in the prevalence of GH excess between expectantly managed patients and asymptomatic patients who underwent surgery (**table 1**), eliminating GH excess as a potential confounder. In an earlier analysis of a subgroup of this population, we had demonstrated an association between increased GH secretion and optic neuropathy [7]. However, in the current analysis of the larger population, there was no longer a statistically significant association between GH excess and vision loss (p = 0.07). We think this represents a change in the patient population due to a change in patient recruitment. Since our earlier publications suggested and found a GH effect on optic neuropathy, we have subsequently been actively recruited young subjects with GH excess and initiated treatment for GH excess as early as possible in an effort to see if long term GH excess morbidity could be averted with treatment. Our most recent analysis of the data on the effect of early detection and treatment of GH excess supports this hypothesis. We were able to show that vision loss in association with GH excess was only seen in subjects whose diagnosis and treatment was delayed until adulthood and that none of the subjects who were diagnosed and treated in childhood had visual disturbances [39].

Optic nerve decompression in the asymptomatic patients is not risk free, as cases of blindness and visual deterioration have been reported [9]. Lei et al attributed a high risk of optic nerve injury to the sensitivity of the already damaged nerve to surgical insult [40]. Three patients in our cohort who underwent simultaneous or sequential prophylactic decompression in the contralateral eye resulted in bilateral blindness. Partial decompression of the optic canal was also suggested as a means for reducing the pressure on the nerve in symptomatic patients. One retrospective review of different surgical techniques and equipment failed to demonstrate any advantage of one particular technology [13]. Thus, risk of nerve injury in asymptomatic patients raises some questions about the actual utility of nerve decompression, especially since disease progression cannot be determined. Furthermore, it is not possible to predict which patient will benefit from the procedure.

### Limitations

There are several limitations related to the design of our current study. First, due to referral bias, there are a higher proportion of patients with MAS in the expectantly managed group than in the FD patient population. Given that MAS is considered a more aggressive variant of the disease, it is conceivable that our population will have a higher risk for ONC. Nevertheless, we did not observe any advantage for carrying out decompression in asymptomatic patients regardless of their GH levels. Second, the retrospective nature of this work precluded our ability to control confounding variables. For example, we did not analyze other features which differed between the operated patients, such as the type of surgery.

### Conclusions

While patients with CFD and radiographic ONC are at potential risk for visual deterioration, most of them are asymptomatic and will remain that way. Surgical decompression should be reserved for symptomatic patients, the majority of whom will show improvement and good long-term results after optic nerve decompression. Expectant management, repeated ophthalmologic exams, and long-term radiologic follow-up are indicated in asymptomatic FD patients who have optic nerve encasement.

## Supporting Information

Table S1 Included studies with prophylactic optic nerve decompression.
(DOC)

Table S2 Included studies with therapeutic optic nerve decompression.
(DOC)

Table S3 Included studies with optic nerve compression observed.
(DOC)

## References

[1] Kelly MH, Brillante B, Collins MT (2008). Pain in fibrous dysplasia of bone: age-related changes and the anatomical distribution of skeletal lesions.. Osteoporos Int.

[2] Weinstein LS, Shenker A, Gejman PV, Merino MJ, Friedman E (1991). Activating mutations of the stimulatory G protein in the McCune-Albright syndrome.. N Engl J Med.

[3] Schwindinger WF, Francomano CA, Levine MA (1992). Identification of a mutation in the gene encoding the alpha subunit of the stimulatory G protein of adenylyl cyclase in McCune-Albright syndrome.. Proc Natl Acad Sci U S A.

[4] McCune DJ, Bruch H (1937). Progress in pediatrics: osteodystrophia fibrosa.. Am J Dis Child.

[5] Albright F, Butler AM, Hampton AO, Smith P (1937). Syndrome characterized by osteitis fibrosa disseminata, areas of pigmentation and endocrine dysfunction, with precocious puberty in females: report of five cases.. New Eng J Med.

[6] Lee JS, FitzGibbon E, Butman JA, Dufresne CR, Kushner H (2002). Normal vision despite narrowing of the optic canal in fibrous dysplasia.. N Engl J Med.

[7] Cutler CM, Lee JS, Butman JA, FitzGibbon EJ, Kelly MH (2006). Long-term outcome of optic nerve encasement and optic nerve decompression in patients with fibrous dysplasia: risk factors for blindness and safety of observation.. Neurosurgery.

[8] Chen YR, Breidahl A, Chang CN (1997). Optic nerve decompression in fibrous dysplasia: indications, efficacy, and safety.. Plast Reconstr Surg.

[9] Edelstein C, Goldberg RA, Rubino G (1998). Unilateral blindness after ipsilateral prophylactic transcranial optic canal decompression for fibrous dysplasia.. Am J Ophthalmol.

[10] Lustig LR, Holliday MJ, McCarthy EF, Nager GT (2001). Fibrous dysplasia involving the skull base and temporal bone.. Arch Otolaryngol Head Neck Surg.

[11] Maher CO, Friedman JA, Meyer FB, Lynch JJ, Unni K (2002). Surgical treatment of fibrous dysplasia of the skull in children.. Pediatr Neurosurg.

[12] Goisis M, Biglioli F, Guareschi M, Frigerio A, Mortini P (2006). Fibrous dysplasia of the orbital region: current clinical perspectives in ophthalmology and cranio-maxillofacial surgery.. Ophthal Plast Reconstr Surg.

[13] Abe T, Satoh K, Wada A (2006). Optic nerve decompression for orbitofrontal fibrous dysplasia: recent development of surgical technique and equipment.. Skull Base.

[14] Panda NK, Parida PK, Sharma R, Jain A, Bapuraj JR (2007). A clinicoradiologic analysis of symptomatic craniofacial fibro-osseous lesions.. Otolaryngol Head Neck Surg.

[15] Cruz AA, Constanzi M, de Castro FA, dos Santos AC (2007). Apical involvement with fibrous dysplasia: implications for vision.. Ophthal Plast Reconstr Surg.

[16] Tan YC, Yu CC, Chang CN, Ma L, Chen YR (2007). Optic nerve compression in craniofacial fibrous dysplasia: the role and indications for decompression.. Plast Reconstr Surg.

[17] Tabrizi R, Ozkan BT (2008). Craniofacial fibrous dysplasia of orbit.. J Craniofac Surg.

[18] Liakos GM, Walker CB, Carruth JA (1979). Ocular complications in craniofacial fibrous dysplasia.. Br J Ophthalmol.

[19] Edgerton MT, Persing JA, Jane JA (1985). The surgical treatment of fibrous dysplasia. With emphasis on recent contributions from cranio-maxillo-facial surgery.. Ann Surg.

[20] Misra M, Mohanty AB, Rath S (1990). Orbital reconstruction in fibrous dysplasia–a case report.. Indian J Ophthalmol.

[21] McCluskey P, Wingate R, Benger R, McCarthy S (1993). Monostotic fibrous dysplasia of the orbit: an unusual lacrimal fossa mass.. Br J Ophthalmol.

[22] Dowler JG, Sanders MD, Brown PM (1995). Bilateral sudden visual loss due to sphenoid mucocele in Albright's syndrome.. Br J Ophthalmol.

[23] Kurimoto M, Endo S, Onizuka K, Akai T, Takaku A (1996). Extradural optic nerve decompression for fibrous dysplasia with a favorable visual outcome.. Neurol Med Chir (Tokyo).

[24] Bocca G, de Vries J, Cruysberg JR, Boers GH, Monnens LA (1998). Optic neuropathy in McCune-Albright syndrome: an indication for aggressive treatment.. Acta Paediatr.

[25] Horgan MA, Delashaw JB, Dailey RA (1999). Bilateral proptosis: an unusual presentation of fibrous dysplasia.. Br J Neurosurg.

[26] Thomas C, Mahapatra AK, Joy MJ, Krishnan A, Sharma RR (2001). Craniofacial surgery in Oman: a preliminary study of 10 cases.. J Craniofac Surg.

[27] Michael CB, Lee AG, Patrinely JR, Stal S, Blacklock JB (2000). Visual loss associated with fibrous dysplasia of the anterior skull base. Case report and review of the literature.. J Neurosurg.

[28] Sharma RR, Mahapatra AK, Pawar SJ, Lad SD, Athale SD (2002). Symptomatic cranial fibrous dysplasias: clinico-radiological analysis in a series of eight operative cases with follow-up results.. J Clin Neurosci.

[29] Fujimoto A, Tsuboi K, Ishikawa E, Nose H, Nose T (2004). Surgery improves vision and cosmetic appearance of an adult patient with fibrous dysplasia of the frontal bone.. J Clin Neurosci.

[30] Movassaghi K, Janecka I (2005). Optic nerve decompression via mid-facial translocation approach.. Ann Plast Surg.

[31] Tajima T, Tsubaki J, Ishizu K, Jo W, Ishi N (2008). Case study of a 15-year-old boy with McCune-Albright syndrome combined with pituitary gigantism: effect of octreotide-long acting release (LAR) and cabergoline therapy.. Endocr J.

[32] Yang X, Guo Z, Mu X, Yu Z (2009). A lateral approach at the upper corner of the orbit in fronto-orbital fibrous dysplasia: less invasive and more effective approach for morphologic reconstruction and optic functional restoration.. J Craniofac Surg.

[33] Bibby K, McFadzean R (1994). Fibrous dysplasia of the orbit.. Br J Ophthalmol.

[34] Liberati A, Altman DG, Tetzlaff J, Mulrow C, Gotzsche PC (2009). The PRISMA statement for reporting systematic reviews and meta-analyses of studies that evaluate health care interventions: explanation and elaboration.. PLoS Med.

[35] Chao K, Katznelson L (2008). Use of high-dose oral bisphosphonate therapy for symptomatic fibrous dysplasia of the skull.. J Neurosurg.

[36] Papay FA, Morales L, Flaharty P, Smith SJ, Anderson R (1995). Optic nerve decompression in cranial base fibrous dysplasia.. J Craniofac Surg.

[37] Kurokawa Y, Sohma T, Tsuchita H, Kitami K, Suzuki S (1989). Hemorrhage into fibrous dysplasia following minor head injury–effective decompression for the ophthalmic artery and optic nerve.. Surg Neurol.

[38] Osguthorpe JD, Gudeman SK (1987). Orbital complications of fibrous dysplasia.. Otolaryngol Head Neck Surg.

[39] Glover M, Kelly MH, Brillante BA, Butman JA, Fitzgibbon EJ (2010). Growth Hormone Excess in McCune-Albright Syndrome: Emphasis on Diagnosis and Treatment in Children.. Endocr Rev Supplement.

[40] Lei P, Bai H, Wang Y, Liu Q (2009). Surgical treatment of skull fibrous dysplasia.. Surg Neurol.

