# Supplementary material for: Surgery versus Watchful Waiting in Patients with Craniofacial Fibrous Dysplasia – a Meta-Analysis
Source: PLoS One. 2011 Sep 23;6(9):e25179. doi: 10.1371/journal.pone.0025179 (PMC3179490; doi:10.1371/journal.pone.0025179)
Supplement: Table S1 — Included studies with prophylactic optic nerve decompression. (DOC) [file pone.0025179.s001.doc]

**Supplementary table S1**

| **Author (year)** | **LOE** | **N** | **OCN** | **Age (years)** | **Follow up (months)** |
| --- | --- | --- | --- | --- | --- |
| Liakos 1979 [31] | C | 1 | 1 | 12 | 132 |
| Bibby 1994 [43] | C | 2 | 2 | 22 | 42 |
| Lustig 2001 [1] | B | 4 | 4 | 26 | 46 |
| Maher 2002 [26] | B | 1 | 1 | N/A | 228 |
| Chen 2007 * | B | 2 | 2 | 23 | 174 |
| Cruz 2007 [29]* | B | 10 | 15 | 14 | 70 |
| Amit 2011* |  | 108 | 216 | 21 | 101 |

**Table S1. Included studies with prophylactic optic nerve decompression (n=41)**
