# Supplementary material for: Surgery versus Watchful Waiting in Patients with Craniofacial Fibrous Dysplasia – a Meta-Analysis
Source: PLoS One. 2011 Sep 23;6(9):e25179. doi: 10.1371/journal.pone.0025179 (PMC3179490; doi:10.1371/journal.pone.0025179)
Supplement: Table S2 — Included studies with therapeutic optic nerve decompression. (DOC) [file pone.0025179.s002.doc]

**Supplementary table S2**

**Table S2. Included studies with therapeutic optic nerve decompression (n=86)**

| **Author (year)** | **LOE** | **N** | **OCN** | **Age (years)** | **Follow up (months)** |
| --- | --- | --- | --- | --- | --- |
| Liakos 1979 [31] | C | 1 | 2 | 20 | 6 |
| Edgerton 1985 [17] | C | 1 | 1 | 18 | 36 |
| Misra 1990 [32] | C | 1 | 1 | 40 | 12 |
| McCluskey 1993 [33] | C | 1 | 1 | 41 | 6 |
| Michel 1994 | C | 1 | 1 | 8 | 6 |
| Dowler 1995 [34] | C | 1 | 2 | 20 | 12 |
| Kurimoto 1996 [19] | C | 1 | 1 | 10 | 36 |
| Chen 1997 [5]* | B | 8 | 9 | 18 | 61 |
| Bocca 199835 | C | 1 | 1 | 17 | 48 |
| Horgan 1999 [36] | C | 1 | 2 | 33 | 12 |
| Thomas 2000 [37] | C | 1 | 1 | 26 | 15 |
| Michael 2000 [7] | C | 1 | 2 | 14 | 17 |
| Maher 2002 [26] | B | 2 | 3 | N/A | 36 |
| Sharma 2002 [38] | C | 2 | 2 | 29 | 51 |
| Fujimoto 2004 [39] | C | 1 | 1 | 31 | 6 |
| Movassaghi 2005 [40] | C | 1 | 2 | 11 | 24 |
| Abe 2006 [25] | B | 1 | 1 | 55 | 84 |
| Goisis 2006 [27]* | C | 1 | 1 | 16 | 92 |
| Chen 2007 [16] | B | 12 | 12 | 19 | 77 |
| Panda 2007* | C | 1 | 1 | 21 | 168 |
| Cruz 2007 [29] | B | 2 | 2 | 20 | 84 |
| Tajima 2008 [41] | C | 1 | 2 | 8 | 84 |
| Yang 2009 [42] | B | 21 | 21 | 26 | 24 |
| Amit 2011* |  | 11 | 14 | 13 | 88 |
