# Supplementary material for: Surgery versus Watchful Waiting in Patients with Craniofacial Fibrous Dysplasia – a Meta-Analysis
Source: PLoS One. 2011 Sep 23;6(9):e25179. doi: 10.1371/journal.pone.0025179 (PMC3179490; doi:10.1371/journal.pone.0025179)
Supplement: Table S3 — Included studies with optic nerve compression observed. (DOC) [file pone.0025179.s003.doc]

**Supplementary table S3**

***Table S3. Included studies with optic nerve compression observed (n=128)***

| **Author (year)** | **LOE** | **N** | **OCN** | **Age (years)** | **Follow up (months)** |
| --- | --- | --- | --- | --- | --- |
| Chen 1997 [5] | B | 4 | 4 | 22 | 45 |
| Edelstein 1998 [23] | C | 1 | 1 | 37 | 6 |
| Lustig 2001 [1] | B | 2 | 2 | 11 | 120 |
| Maher 2002 [26] | B | 3 | 3 | N/A | 162 |
| Goisis 2006 [27] | C | 3 | 4 | 15 | 88 |
| Abe 2006 [28] | B | 5 | 5 | 19 | 54 |
| Panda 2007 [14] | C | 1 | 1 | 21 | 168 |
| Cruz 2007 [29] | B | 2 | 2 | 16 | 54 |
| Chen 2007 [16] | B | 6 | 6 | 22 | 94 |
| Tabrizi 2008 [30] | C | 3 | 3 | 19 | 22 |
| Amit 2011 |  | 8 | 10 | 20 | 114 |

LOE = level of evidence, MAS = McCune Albright’s syndrome, OCD = optic canal decompression, OCN = optic canal narrowing, N/A = not available.

Continuous variables are presented as means.
